# Usage of PCI and long-term cardiovascular risk in post-myocardial infarction patients: a nationwide registry cohort study from Finland

**DOI:** 10.1186/s12872-019-1101-8

**Published:** 2019-05-22

**Authors:** Ville Kytö, Tuire Prami, Houssem Khanfir, Pål Hasvold, Eeva Reissell, Juhani Airaksinen

**Affiliations:** 1 Turku University Hospital and University of Turku, Heart Center, PO Box 52, 20521 Turku, Finland; 2 Research Centre of Applied and Preventive Cardiovascular Medicine, University of Turku, Turku, Finland; 3 EPID Research, Espoo, Finland; 4 AstraZeneca Nordic Baltic, Södertälje, Sweden; 5 National Institute for Health and Welfare, Helsinki, Finland

**Keywords:** Myocardial infarction, PCI, Survival, Epidemiology

## Abstract

**Background:**

Despite currently available treatments, the burden of myocardial infarction (MI) morbidity and mortality remains prominent. The aim of this was to investigate the risk of developing subsequent cardiovascular events in MI patients.

**Methods:**

This was an observational, retrospective cohort database linkage study using patient level data from Finland. Cox proportional hazards models were used to assess the association of risk between the preselected covariates and incidence of specific outcomes. The primary endpoints were new MI, stroke, cardiovascular mortality and overall mortality.

**Results:**

Finnish adult MI patients alive 7 days after discharge in 2009**–**2012 were included. The study cohort consisted of 32,909 MI patients, of whom 25,875 (79%) survived 12 months without subsequent MI or stroke. ST-elevation MI (STEMI) was associated with lower risk of subsequent MI and overall mortality compared to non-STEMI patients. Percutaneous coronary intervention (PCI) was used two times more often in STEMI patients, but patients with prior stroke were more than two times less likely to have PCI. Dementia/Alzheimer’s disease decreased the use of PCI as much as age over 85 years. Female sex was an independent factor for not undergoing PCI (OR 0.75, *P* < 0.001 compared to men) but was nevertheless associated with lower risk of new MI and mortality (HR 0.8–0.9, *P* < 0.001 for all). Increased age was associated with increased event risk and PCI with decreased event risk.

**Conclusions:**

Risk of cardiovascular events and mortality after MI increases steeply with age. Although at higher risk, aging patients and those with cardiovascular comorbidities are less likely to receive PCI after MI. Female sex is associated with better survival after MI regardless of less intensive treatment in women.

**Electronic supplementary material:**

The online version of this article (10.1186/s12872-019-1101-8) contains supplementary material, which is available to authorized users.

## Background

Myocardial infarction (MI) causes significant morbidity, and ischemic heart disease is the leading cause of death worldwide [1]. Developments and increasing use of treatment modalities, namely pharmacotherapy and invasive interventions, have improved the outcomes after MI in recent years [2, 3]. Due to shifts in population demographics, the burden of coronary artery disease (CAD)-caused mortality and morbidity will, however, continue to have a major impact in the foreseeable future [4, 5].

Improved outcome after MI is increasing the proportion of stable post-MI CAD patients [3] who are, however, at high risk of subsequent cardiovascular events [5–7]. In many countries, including Finland, the long-term follow-up of post-MI patients is largely handled in primary care, without direct contact with a specialist. In an environment of limited health care resources, identification of factors associated with a high risk of adverse events in post-MI patients is essential for improving treatment and follow-up strategies after MI. This information is however currently limited. In the PEGASUS-TIMI 54 trial, 9.0% of stable patients enrolled 1–3 years post-MI had a major adverse cardiovascular event (MACE) during the 3-year follow-up when not on dual antiplatelet therapy (DAPT) [8]. A nationwide Swedish registry study of post-MI patients found the corresponding MACE rate to be 20% [6]. Aging is associated with worse outcome in the post-MI population, as is the presence of diabetes, heart failure and multiple cardiovascular events [9].

Use of secondary preventive medication improves outcomes after MI. Long-term DAPT with ticagrelor reduced the MACE rate in high-risk post-MI patients by 15–16% during the 3-year follow-up [8]. In real-world clinical practice, however, the use of DAPT is notably suboptimal, with a recent large-scale registry from Finland showing that only half of acute coronary syndrome patients received DAPT after MI [10]. Percutaneous coronary intervention (PCI) as primary PCI in ST-elevation MI (STEMI) or as an urgent procedure in non-ST-elevation MI (NSTEMI) significantly improves survival and subsequent events, with high-risk patients experiencing the most benefit [11, 12]. Individual level predictors of PCI use in MI are, however, less well known.

The aim of this real-world nationwide study was to examine the outcome and its determinants after MI, with a focus on patients surviving more than a year after MI without a major cardiovascular. Furthermore, we studied factors associated with the use of PCI in MI.

## Methods

In this observational, retrospective cohort study, data from different nationwide administrative health care registers, including information on hospitalizations, diagnoses, outpatient drug use and causes of death, were used [10]. Medication data were based on Anatomical Therapeutic Chemical (ATC) codes. Cause-specific mortality outcomes were identified using data for the primary cause of death only; for the other outcome events, both primary and secondary diagnoses were used to identify the outcomes of interest.

The study population consisted of Finnish adult patients discharged from Finnish hospitals following admission for MI (International Classification of Diseases, Tenth Revision [ICD-10] code: I21) between 01 January 2009 and 31 December 2012 and alive 7 days after the discharge. Patients hospitalized in the autonomic Aland Islands were excluded.

**Fig. 1:**
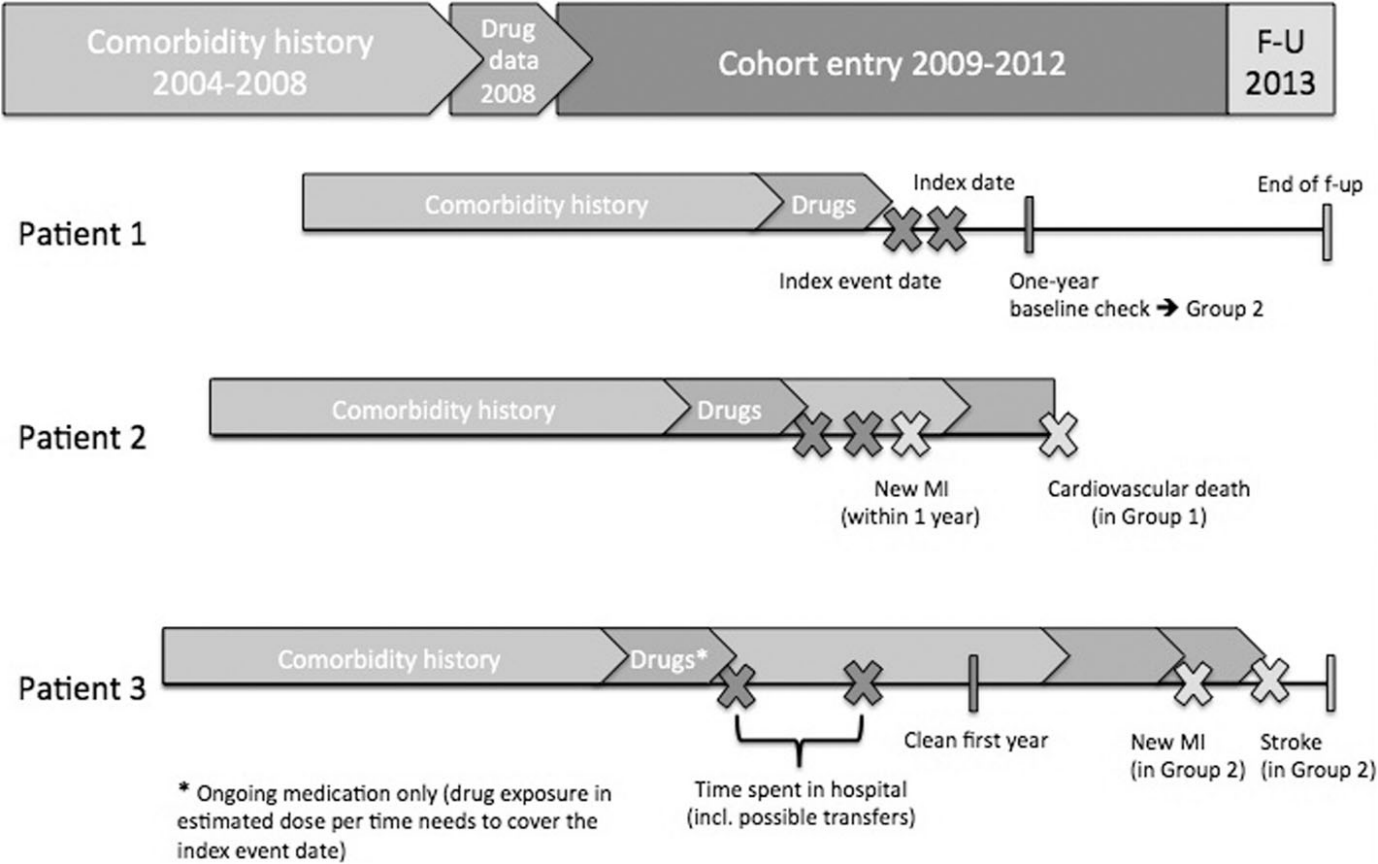
Definitions related to study design with example study patient cases

Index event was admission due to MI, and discharge day was the index date (Fig. 1). In case of hospital transfer(s) after the index event (patients with discharge and new hospitalization on the same day), the index date was the last discharge date after the transfer(s). Medical disease history was evaluated from 5 years before the index date, and medication history from 1 year before the index date for each study patient separately (Fig. 1). The latter was included in an assessment of medication ongoing at the index event, and this information was used as a proxy for diabetes mellitus, hypertension and hyperlipidemia, and to differentiate between new and ongoing users of secondary preventive medications. Patients with a history of MI before the study period were excluded.

The primary outcomes were hospitalizations due to MI (ICD-10: I21–I22) and ischemic stroke (ICD-10: I63), cardiovascular mortality (ICD-10: I21–I22, I61–I64, I48, I49, I50), and overall mortality. The whole study cohort was labelled as Group 1, and the stable post-MI population without any recurrent MI or stroke during the first year after index MI as Group 2. For Group 1, follow-up began at the index date, and for Group 2, follow-up time began at the 1-year baseline check. For both groups, the follow-up ended at the time of death, moving abroad or at the end of study period (December 31, 2013), whichever occurred first (Fig. 1). Furthermore, for a particular endpoint, the follow-up ended at the time of the first occurrence of the endpoint but continued for the other endpoints. In the results, the baseline characteristics are presented also for those patients who experienced an endpoint during the first year (Group 1b).

The odds ratio of undergoing PCI related to the index event (PCI between admission and index date) was modelled using logistic regression. In this multivariate test, predictor variables included age, sex and type of index MI, as well as history of atrial fibrillation, diabetes mellitus, chronic renal failure, dementia/Alzheimer’s disease, ischemic stroke or transient ischemic attack (TIA), major bleedings, hypertension, hyperlipidemia, congestive heart failure, severe liver disease, chronic obstructive pulmonary disease (COPD) and malignancy. Variable definitions for diabetes mellitus, hypertension and hyperlipidemia included disease-specific drug use as proxy (ATC groups A10; C10; C02, C03, C07, C08 and C09, respectively). Confidence intervals (CIs) for the odds ratios and *P* values were also reported.

In order to assess incidence rates for the primary outcomes, non-parametric estimates of cumulative incidence and stratified incidence rates with 95% CIs were estimated. Cumulative incidence rates were calculated accounting for deaths due to other causes than the outcome of interest as competing risk events. The 95% CIs were derived under the Poisson assumption.

Cox proportional hazards models were used to assess the association of risk between the preselected covariates and incidence of specific outcomes. In this multivariate model, the results were adjusted by age, sex, type of index MI, and PCI or coronary artery bypass grafting (CABG) related to index event, as well as by the following time-dependent comorbidity variables: atrial fibrillation, diabetes mellitus, chronic renal failure, dementia/Alzheimer’s disease, ischemic stroke or TIA, major bleedings, hypertension, hyperlipidemia, congestive heart failure, severe liver disease, COPD, malignancy, ongoing selective serotonin reuptake inhibitor (SSRI) use, and ongoing oral antiplatelet (OAP: clopidogrel, prasugrel or ticagrelor) use. Time after admission to institutional care, such as elderly home care, was censored from the follow-up in this model, as the information on drug treatments was not available. Cohort entry years were used as strata in the model.

For exploratory outcomes, the primary outcomes were sub-classified into specific causes, and the non-parametric estimates of cumulative incidence of each sub-cause were presented. For the mortality outcome, the 5 most common causes of death were identified. The R language [13] was used for data management and all statistical modeling.

## Results

**Fig. 2:**
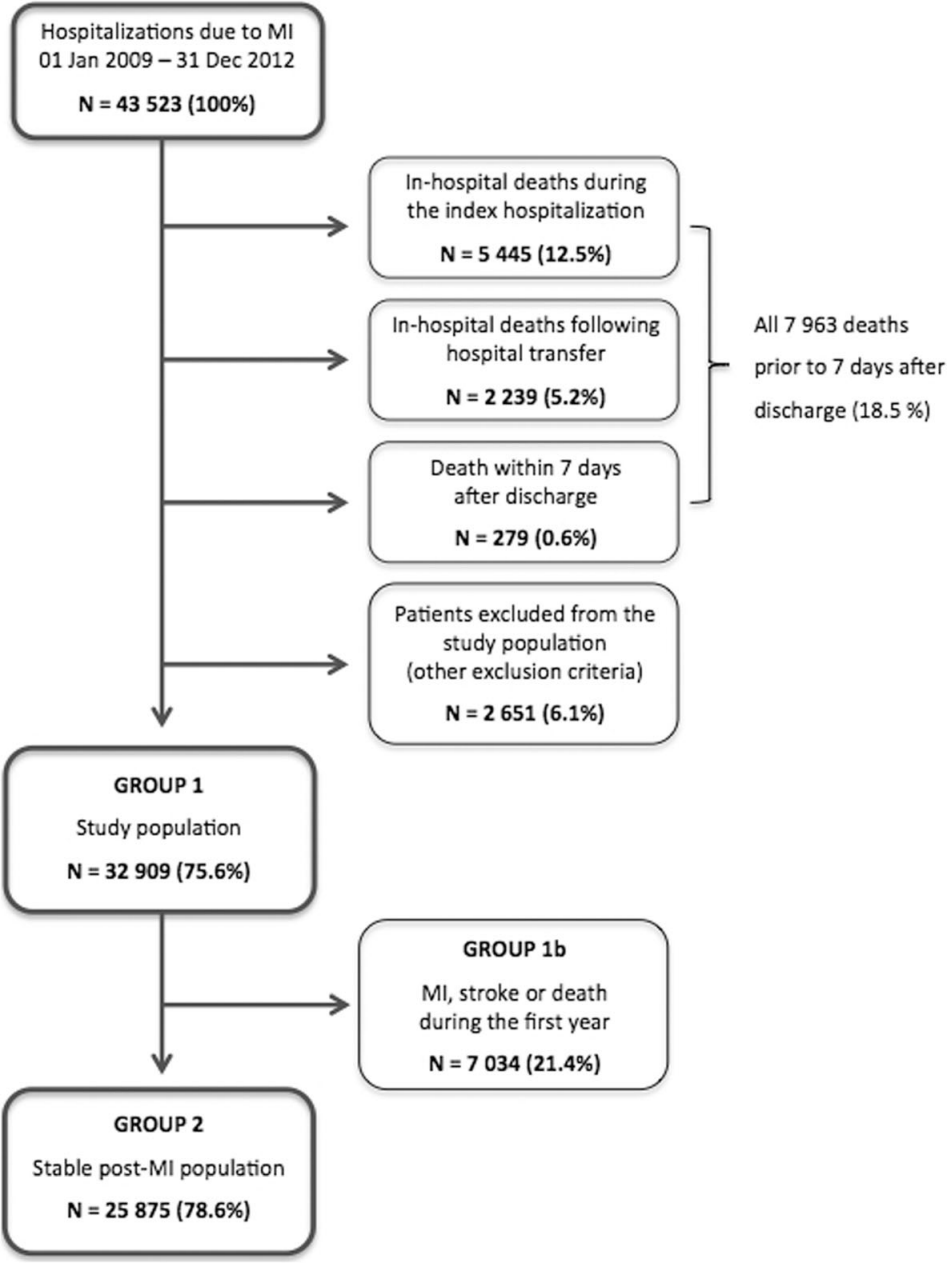
Population flow chart

During the study period, 43,523 patients were admitted to hospital due to MI, of whom 32,909 were included in the study cohort, i.e. Group 1 (Fig. 2). Of these, 25,875 (79%) survived 12 months without subsequent MI or stroke (Group 2). Mean age was 72 years, and 61% of the patients in the study cohort were men (Table 1). NSTEMI was the index event in 66% of the patients. Those patients who experienced a subsequent cardiovascular event or death during the first year of follow-up (Group 1b) were more often NSTEMI patients, not treated invasively, older, and had more underlying diseases.

**Table 1:** Index event and patient baseline characteristics^a^

	Group 1	Group 1 b	Group 2
N of patients	32,909	7034	25,875
Follow-up duration (years) mean (±sd)	2.48 (1.31)	1.42 (1.34)	1.78 (1.14)
Type of index MI			
STEMI	11,270 (34.25%)	1742 (24.77%)	9528 (36.82%)
NSTEMI (or unspecified MI)	21,639 (65.75%)	5292 (75.23%)	16,347 (63.18%)
Interventions related to index MI			
No invasive procedure	13,793 (41.91%)	4697 (66.78%)	9096 (35.15%)
Coronary angiography only	4702 (14.29%)	772 (10.98%)	3930 (15.19%)
PCI	12,203 (37.08%)	1353 (19.24%)	10,850 (41.93%)
CABG	2211 (6.72%)	212 (3.01%)	1999 (7.73%)
Age (years)			
mean (±sd)	71.98 (12.97)	78.23 (11.43)	71.27 (12.84)
range (min; max)	18.52; 102.83	27.85; 102.83	19.50; 103.12
Sex			
Male	19,962 (60.66%)	16,116 (62.28%)	3846 (54.68%)
Female	12,947 (39.34%)	9759 (37.72%)	3188 (45.32%)
Prior cardiovascular comorbidities			
Atrial fibrillation	4758 (14.46%)	1549 (22.02%)	3657 (14.13%)
Major bleedings	1879 (5.71%)	635 (9.03%)	1632 (6.31%)
Ischemic stroke or TIA	2679 (8.14%)	979 (13.92%)	1575 (6.09%)
Congestive heart failure	6413 (19.49%)	2426 (34.49%)	4798 (18.54%)
Hypertension	29,942 (90.98%)	6533 (92.88%)	25,339 (97.93%)
Hyperlipidemia	24,104 (73.24%)	4542 (64.57%)	22,875 (88.41%)
Other prior comorbidities			
Diabetes mellitus	8256 (25.09%)	2304 (32.76%)	6601 (25.51%)
Chronic renal failure	719 (2.18%)	329 (4.68%)	500 (1.93%)
Dementia/Alzheimer’s disease	1591 (4.83%)	658 (9.35%)	1207 (4.66%)
Severe liver disease	98 (0.30%)	37 (0.53%)	84 (0.32%)
COPD	1224 (3.72%)	415 (5.90%)	917 (3.54%)
Malignancy	2241 (6.81%)	828 (11.77%)	1647 (6.37%)
Index year			
2009	7885 (23.96%)	1750 (24.88%)	6135 (23.71%)
2010	8062 (24.50%)	1732 (24.62%)	6330 (24.46%)
2011	8426 (25.60%)	1760 (25.02%)	6666 (25.76%)
2012	8536 (25.94%)	1792 (25.48%)	6744 (26.06%)

^a^Patient characteristics for Group 1 and Group 1b at index date, and for Group 2 at 1-year baseline check. Follow-up duration of all patients

Abbreviations: *CABG* coronary artery bypass grafting, *COPD* chronic obstructive pulmonary disease, *MI* myocardial infarction, *NSTEMI* non-ST-elevation myocardial infarction, *PCI* percutaneous coronary intervention, *sd* standard deviation, *STEMI* ST-elevation myocardial infarction, *TIA* transient ischemic attack

Of all included MI patients, 37% underwent PCI at the time of index event. When stratified by age, 47% of patients younger than 80 years of age were treated with PCI, compared with only 16% of patients 80 years and older. Of STEMI patients, 55% underwent PCI in comparison to 28% of NSTEMI patients. Women were treated less often with PCI than men (26% vs 44%). When stratifying by both sex and type of index MI, between-sex difference was still present; 60% of male STEMI patients had PCI, but only 44% of female patients with STEMI were treated with PCI. Multivariate-adjusted odds ratios for predictors of undergoing PCI are shown in Table 2. Female sex was an independent predictor for not undergoing PCI, with 25% smaller odds than males (Table 2). Dementia/Alzheimer’s disease diagnosis independently decreased the probability of PCI as much as age over 85 years. Of the stable post-MI population, 42% underwent PCI at the time of the index event (Table 1).

**Table 2:** Adjusted odds ratios for predictors of undergoing PCI related to index event

	OR	95% CI	P
Age (vs < 50)			
50–64	0.862	0.774–0.959	0.006
65–69	0.680	0.603–0.767	< 0.001
70–74	0.670	0.595–0.754	< 0.001
75–79	0.544	0.483–0.613	< 0.001
80–84	0.424	0.375–0.479	< 0.001
85 and over	0.179	0.156–0.205	< 0.001
Female sex (vs male)	0.745	0.705–0.788	< 0.001
STEMI as index event (vs NSTEMI)	2.281	2.165–2.402	< 0.001
Atrial fibrillation	0.746	0.686–0.811	< 0.001
Diabetes mellitus	0.777	0.732–0.825	< 0.001
Chronic renal failure	0.585	0.477–0.718	< 0.001
Dementia/Alzheimer’s disease	0.195	0.153–0.247	< 0.001
Ischemic stroke or TIA	0.467	0.417–0.523	< 0.001
Major bleedings	0.565	0.496–0.643	< 0.001
Hypertension	0.833	0.758–0.915	< 0.001
Hyperlipidemia	1.886	1.760–2.022	< 0.001
Congestive heart failure	0.527	0.488–0.569	< 0.001
Severe liver disease	0.739	0.444–1.231	0.245
COPD	0.582	0.501–0.677	< 0.001
Malignancy	0.691	0.618–0.771	< 0.001

Abbreviations: *CI* confidence interval, *COPD* chronic obstructive pulmonary disease, *NSTEMI* non-ST-elevation myocardial infarction, *OR* odds ratio, *P* probability, *STEMI* ST-elevation myocardial infarction, *TIA* transient ischemic attack

The multivariate model simultaneously included all the variables listed in this table

A beta-blocker was the most commonly used secondary preventive drug after MI (Table 3). A lower proportion of patients with a cardiovascular event or death during the first year following MI (Group 1b) was treated with recommended secondary preventive drugs, whereas in the stable post-MI population, 60% of patients used a minimum of three out of the four recommended drugs after discharge (Table 3).

**Table 3:** Secondary preventive drug use at discharge^a^

	Group 1	*Group 1 b*	Group 2
N of patients	32,909	7034	25,875
OAP	17,056 (51.8%)	2318 (33.0%)	14,738 (57.0%)
Beta-blocker	24,119 (73.3%)	4341 (61.7%)	19,778 (76.4%)
Statin	22,123 (67.2%)	3387 (48.2%)	18,736 (72.4%)
ACE inhibitor or ARB	18,663 (56.7%)	3167 (45.0%)	15,496 (59.9%)
Number of secondary preventive drugs			
0	4221 (12.8%)	1644 (23.4%)	2577 (10.0%)
1	4284 (13.0%)	1428 (20.3%)	2856 (11.0%)
2	6497 (19.7%)	1548 (22.0%)	4949 (19.1%)
3	9069 (27.6%)	1434 (20.4%)	7635 (29.5%)
4	8838 (26.9%)	980 (13.9%)	7858 (30.4%)
mean (+/−sd)	2.4 (1.3)	1.8 (1.4)	2.6 (1.3)
range (min; max)	0.0; 4.0	0.0; 4.0	0.0; 4.0
median (Q1; Q3)	3.0 (1.0; 4.0)	2.0 (1.0; 3.0)	3.0 (2.0; 4.0)

^a^(prescription filled within 30 days for new users and within 90 days for old users)

Abbreviations: ACE, angiotensin-converting enzyme; ARB angiotensin receptor blocker; OAP, oral antiplatelet; Q, quartile; sd, standard deviation

**Fig. 3:**
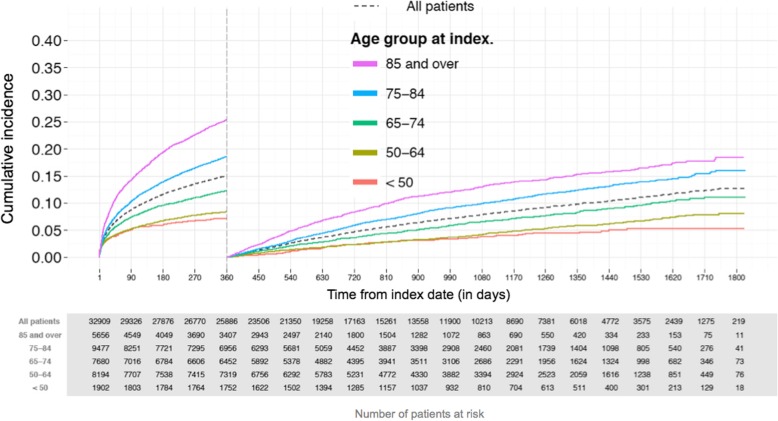
Cumulative incidence of major cardiovascular events (including myocardial infarction, ischemic stroke or cardiovascular mortality) stratified by age categories in Group 1 (for the first year) and for Group 2 (starting from day 360 after index date)

**Fig. 4:**
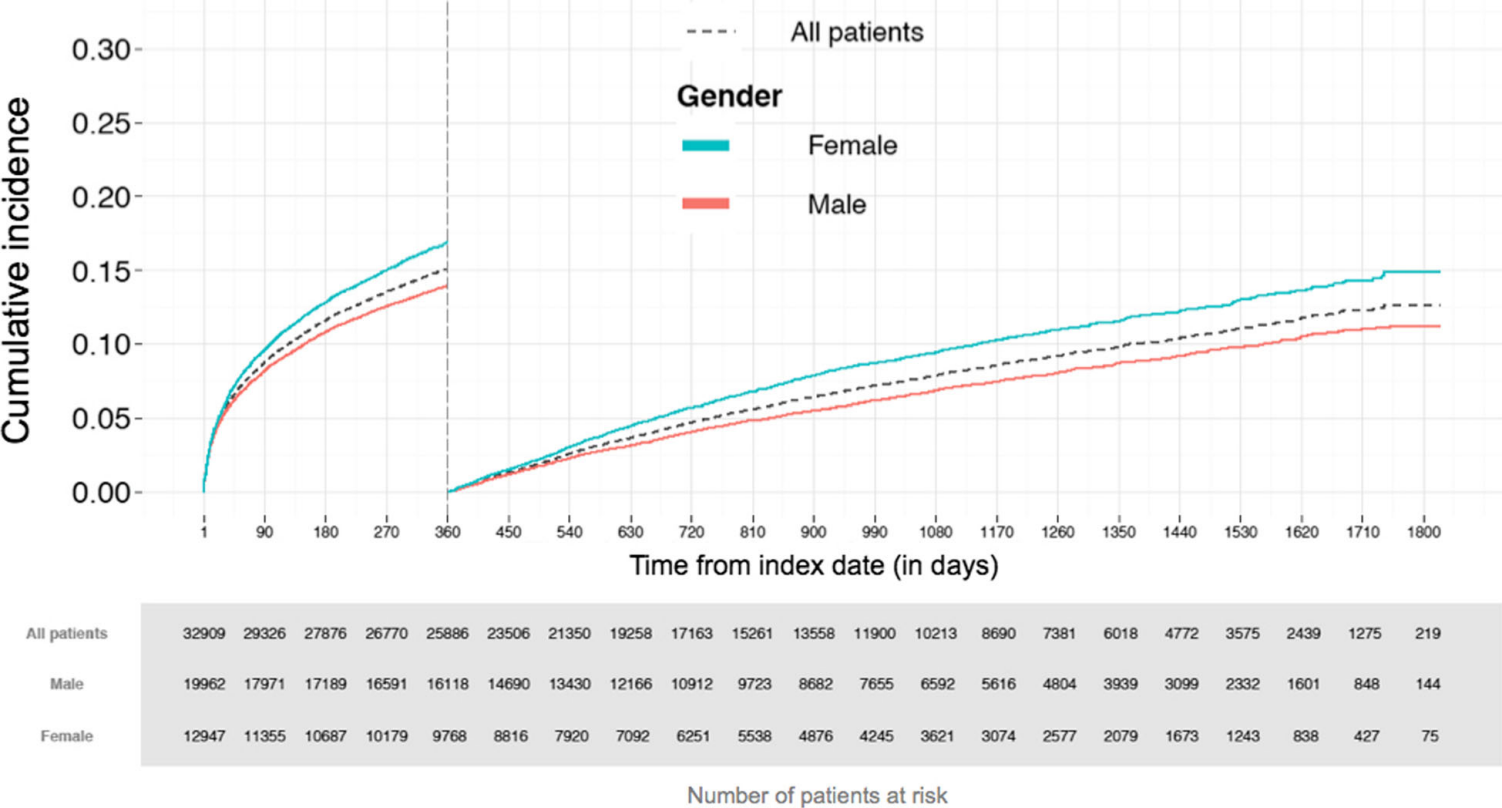
Cumulative incidence of major cardiovascular events (including myocardial infarction, ischemic stroke or cardiovascular mortality) stratified by gender in Group 1 (for the first year) and for Group 2 (starting from day 360 after index date)

Occurrence of MACE, pooling MI, ischemic stroke or cardiovascular mortality, increased rapidly early after index MI in all age categories. The cumulative MACE rate after initial increase was strongly associated with increasing age (Fig. 3). In patients aged < 65 years, the MACE rate remained under 10% for the first year of follow-up, but in older patients, the likelihood of MACE increased progressively, with a rate of over 25% at 1 year in patients aged 85 years or older. On the other hand, for those who survived the first year of follow-up without outcome events, the cumulative risk of MACE was less than 10% in all age categories for the following year (at 2 years following the index MI), with 5% risk overall. In unadjusted analyses, women had a greater risk of MACE than men during the first follow-up year and thereafter (Fig. 4). Women were, however, significantly older than men (mean age 77.4 years vs 68.5 years, *P* < 0.001).

Multivariate-adjusted hazard ratios (HRs) for the primary outcomes with respect to patient characteristics and comorbidities for the total study population are presented in Table 4. The HRs from the full statistical model, including the stable post-MI population, are given in Additional file 1: Table S1. Risk factors for cardiovascular events in the total cohort and in the stable post-MI population were similar. Older age was associated with a higher risk of all adverse outcomes and invasive treatment with a lower risk. STEMI patients had lower risk of new MI and overall mortality compared to NSTEMI patients. Female sex was associated with a lower risk of MI (HR 0.89, *P* < 0.001), cardiovascular mortality (HR 0.79, *P* = 0.002) and overall mortality (HR 0.78, *P* < 0.001) in Group 1, and with a lower risk of MI (HR 0.88, *P* = 0.025) and overall mortality (HR 0.80, *P* < 0.001) during the stable post-MI period (in Group 2) when accounting for age and comorbidities. History of ischemic stroke or TIA was associated with increased risk of all outcomes, stroke in particular (3- to 4-fold, *P* < 0.001). Diabetes was also associated with increased risk of all outcomes. Atrial fibrillation was associated with a 40–50% risk increment of stroke (*P* < 0.001). Dementia/Alzheimer’s disease, congestive heart failure and COPD were associated with approximately doubled risk of overall mortality (*P* < 0.001). History of major bleeding events increased this risk by 35% (*P* < 0.001) in the total cohort. We found that ongoing OAP treatment was associated with a 33% (*P* < 0.001) lower risk of overall mortality in the total population. Stable post-MI patients treated with an OAP after the 12 months had recurrent MI more commonly than patients without need for extended OAP therapy.

**Table 4:** Adjusted hazard ratios for predictors of primary outcomes in total study cohort (Group 1)

	Myocardial infarction		Ischemic stroke		Cardiovascular mortality		Overall mortality	
	HR	P	HR	P	HR	P	HR	P
Age (vs < 50)								
50–64	1.018	0.841	2.058	0.003	2.013	0.078	2.020	< 0.001
65–69	1.309	0.004	2.690	< 0.001	2.162	0.063	2.250	< 0.001
70–74	1.308	0.003	3.229	< 0.001	3.572	0.001	2.962	< 0.001
75–79	1.676	< 0.001	3.736	< 0.001	4.804	< 0.001	3.736	< 0.001
80–84	2.038	< 0.001	4.476	< 0.001	5.160	< 0.001	4.251	< 0.001
85 and over	2.745	< 0.001	4.953	< 0.001	8.894	< 0.001	6.476	< 0.001
Female sex (vs male)	0.887	< 0.001	0.905	0.085	0.789	0.002	0.782	< 0.001
STEMI as index event (vs NSTEMI)	0.881	< 0.001	1.118	0.074	0.842	0.066	0.843	< 0.001
PCI or CABG related to index MI	0.650	< 0.001	0.673	< 0.001	0.502	< 0.001	0.493	< 0.001
Atrial fibrillation	1.014	0.725	1.408	< 0.001	1.057	0.528	1.143	0.003
Diabetes mellitus	1.371	< 0.001	1.257	< 0.001	1.494	< 0.001	1.361	< 0.001
Chronic renal failure	1.788	< 0.001	1.310	0.062	1.265	0.193	1.762	< 0.001
Dementia/Alzheimer’s disease	0.979	0.726	0.969	0.768	2.029	< 0.001	2.541	< 0.001
Ischemic stroke or TIA	1.114	0.017	4.169	< 0.001	1.601	< 0.001	1.332	< 0.001
Major bleedings	1.170	0.002	1.144	0.126	1.243	0.052	1.352	< 0.001
Hypertension	0.910	0.332	1.319	0.245	0.659	0.052	0.582	< 0.001
Hyperlipidemia	1.016	0.701	0.974	0.745	0.721	< 0.001	0.616	< 0.001
Congestive heart failure	1.385	< 0.001	1.219	0.002	2.000	< 0.001	2.018	< 0.001
Severe liver disease	1.232	0.353	1.870	0.061	0.721	0.645	1.283	0.381
COPD	1.156	0.032	0.950	0.706	1.842	< 0.001	1.915	< 0.001
Malignancy	1.160	0.003	1.272	0.006	0.975	0.841	1.668	< 0.001

Abbreviations: *CABG* coronary artery bypass grafting, *MI* myocardial infarction, *NSTEMI* non-ST-elevation myocardial infarction, *PCI* percutaneous coronary intervention, *STEMI* ST-elevation myocardial infarction, *TIA* transient ischemic attack

The multivariate model simultaneously included all the variables listed in this table and in addition ongoing selective serotonin reuptake inhibitor use, and ongoing oral antiplatelet use

The full statistical model for Group 1 and Group 2 for all outcomes is presented in Additional file 1: Table S1

**Fig. 5:**
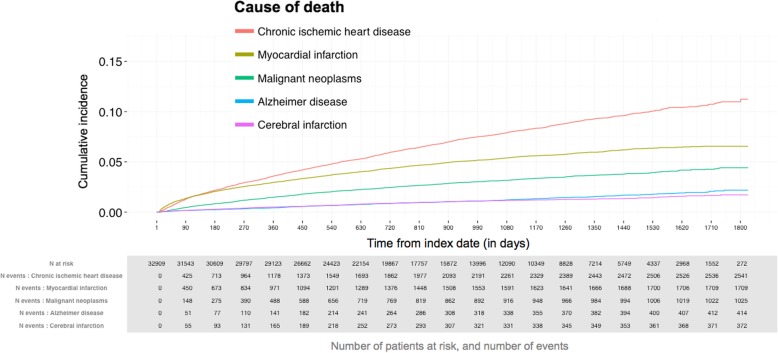
Cumulative incidence of the most common causes of death in the study population (Group 1). The follow-up is from index date to the end of study

Ischemic heart disease was the most common cause of death during follow-up (Fig. 5), with the proportion of patients dying from acute MIs decreasing over time in relation to chronic manifestations of ischemic heart disease.

## Discussion

This population-based real-world registry study shows that one out of five of all MI patients had a subsequent MI or ischemic stroke or died during the first year of follow-up. Cardiovascular risk persisted thereafter, as one out of 20 patients without a cardiovascular event during the first year after index MI experienced a MACE by the second year post index MI. This long-term risk was independently associated with aging, diabetes, heart failure and history of stroke or TIA. STEMI was associated with lower risk of subsequent MI. PCI was used significantly less often in female patients. Nevertheless, female sex was associated with lower risk of new MI and mortality. Important risk factors for cardiovascular events were similar across the overall and stable post-MI patient populations.

Not surprisingly, older age was a major predictor of all cardiovascular outcomes. The risk of MI doubled by the age of 80 years compared to that in patients < 50 years old. The effect of aging was, however, even more prominent in the risks of stroke and death, which were doubled already by the age of 50–64 years. In agreement with previous findings [6, 14, 15], we found the risk of subsequent events to be highest early after MI. Cumulative incidence of MACE increased to 5% in every age category within 90 days of the index date, after which it remained < 10% in the youngest patients but increased to 25% in the oldest patients during the first year of follow-up (Fig. 3).

In the stable post-MI population, even the oldest patients remained, however, at a MACE risk level of < 10% for the whole second year following index MI (Fig. 3). Jernberg et al. have published a similar risk estimates, but in their cohort, patients aged > 80 years had an almost 20% risk at the same time point [6]. Furthermore, at 4 years of follow-up, the risk of the composite endpoint was < 20% in our setting but > 40% in the Swedish setting in the oldest age category. However, a lower in-hospital mortality was observed in Sweden, and the exclusion of patients with a history of prior MI in the present survey may contribute to this difference.

Importantly, the OAP users had an over 50% lower mortality risk than non-OAP users during the first month after the index MI (*P* < 0.001). This, together with the result that only 52% of the MI patients in our nationwide cohort started the OAP medication after the MI, highlights the importance of encouragement and assurance of the guideline-recommended [16, 17] medication initiation.

In general, the use of secondary preventive medication immediately after MI has been found to be high in the Nordic countries [10, 14, 15, 18]. The proportion of all MI patients treated with statins and OAPs is, however, somewhat lower in Finland. Compliance with initiated secondary preventive medication was good in the Finnish stable post-MI population, though, with more than 70% of patients using a beta-blocker and a statin in our study in Group 2 after discharge.

In our study population, one fifth of stable post-MI patients were using OAP therapy (94% clopidogrel) beyond 12 months [16, 17]. These patients had higher rates of MIs in spite of the prolonged antithrombotic therapy. This finding probably reflects selection bias but suggests that the high-risk patients may need more effective antithrombotic treatment options to prevent reinfarction. Previously, the CHARISMA trial of long-term clopidogrel treatment in stable patients with atherosclerotic disease did not find treatment benefit [19]. Post-hoc analysis of the CHARISMA subpopulation did, however, indicate benefit in patients with prior MI, stroke or peripheral artery disease [20]. The PEGASUS-TIMI 54 trial showed that low-dose ticagrelor treatment reduced MACE events in high-risk patients beyond 1 year after MI [8].

Diabetes was an important comorbidity, associated with a 25–55% higher risk of all primary outcomes in all study patients and also in stable post-MI patients. Dementia/Alzheimer’s disease, heart failure and COPD doubled the risk of cardiovascular and overall mortality. Both dementia/Alzheimer’s disease and age over 85 years decreased the use of PCI more than 80%. Invasive treatment as such significantly (30–50%, *P* < 0.001) protected against all outcome events in both Group 1 and Group 2.

In relation to subsequent MIs, STEMI patients had a better prognosis in the whole cohort, especially in the stable post-MI population, than NSTEMI patients. Our study supports the recent finding that MI type does not have an effect on the risk of stroke [21]. PCI has been reported to be used more commonly in STEMI [22], which was seen also in our data.

Previous studies have reported women to be at higher short-term and long-term mortality risk after MI [23–28]. Accordingly, we found women to have a higher unadjusted MACE rate after MI. This result was, however, driven by the fact that women were significantly older than men at the time of the index MI. Previous studies have shown that men receive cardiac rehabilitation [29, 30] and guideline-based pharmacotherapy after MI more often than women [10]. In the present study, we found women to be much less likely to receive PCI than men. Surprisingly, in spite of this treatment difference, female sex was associated with lower rate of subsequent MI, cardiovascular mortality and overall mortality in the whole cohort, and with lower rate of subsequent MI and overall mortality in the stable post-MI population when accounting for age and comorbidities. This may at least partly be related to reasonable assumption that in women, the recorded MIs more often include lower-risk events e.g. Takotsubo cardiomyopathy and Type 2 MIs.

The nature of the study has some inherent limitations. The data were not originally collected for study purposes but originated from administrative registers. The source registers are based on diagnoses and operational codes registered by treating physicians and do not include detailed clinical information about MI patients, such as type of stents used in PCIs or results of laboratory measurements. Diagnoses of multivessel CAD are underreported, and information on smoking is not included in these care registers. Our study has a nationwide coverage without social or insurance statuses affecting the patient selection. Survival bias may, however, have affected the analyses in stable post-MI patients. Exact drug doses were not available in the data, but careful definition of OAP exposure was possible due to uniform dosing. The use of other drugs was measured in defined daily doses (DDDs), and drug exposures were handled time-dependently. Study size was sufficiently powered to detect single outcomes. We did, however, also run analyses in pooled MACE to be able to compare the results with similar studies.

## Conclusions

In conclusion, risk of cardiovascular events and mortality after MI increases steeply with aging. PCI/CABG was clearly protective against subsequent MACE and overall mortality. Although at higher risk, aging patients and those with cardiovascular comorbidities are less likely to receive PCI after MI. Female sex appears to promote protection from subsequent events and to increase survival after MI regardless of less use of PCI in women.

## Additional file

Additional file 1: **Table S1.** Adjusted hazard ratios for predictors of primary outcomes in total study and stable post-MI populations. (DOCX 115 kb)

## Abbreviations

ATC: Anatomical therapeutic chemical
CABG: Coronary artery bypass grafting
CAD: Coronary artery disease
CIs: Confidence intervals
COPD: Chronic obstructive pulmonary disease
DAPT: Dual antiplatelet therapy
ICD-10: International classification of diseases, tenth revision
MACE: Major adverse cardiovascular event
MI: Myocardial infarction
NSTEMI: Non-ST-elevation MI
OAP: Oral antiplatelet
PCI: Percutaneous coronary intervention
SSRI: Selective serotonin reuptake inhibitor
STEMI: ST-elevation MI
TIA: Transient ischemic attack
