# Supplementary material for: Usage of PCI and long-term cardiovascular risk in post-myocardial infarction patients: a nationwide registry cohort study from Finland
Source: BMC Cardiovasc Disord. 2019 May 22;19:123. doi: 10.1186/s12872-019-1101-8 (PMC6532224; doi:10.1186/s12872-019-1101-8)
Supplement: Supplementary file 1 — Table S1. Adjusted hazard ratios for predictors of primary outcomes in total study and stable post-MI populations. (DOCX 115 kb) [file 12872_2019_1101_MOESM1_ESM.docx]

**Additional file 1: Table S1.** Adjusted hazard ratios for predictors of primary outcomes in total study (Group 1) and stable post-MI (Group 2) populations

|  | **Myocardial infarction** | | | | **Ischemic stroke** | | | | **Cardiovascular mortality** | | | | **Overall mortality** | | |
| --- | --- | --- | --- | --- | --- | --- | --- | --- | --- | --- | --- | --- | --- | --- | --- |
|  | **Group 1** | | **Group 2** | | **Group 1** | | **Group 2** | | **Group 1** | | **Group 2** | | **Group 1** | | **Group 2** |
| Age (vs <50) |  | |  | |  | |  | |  | |  | |  | |  |
| 50-64 | 1.018 | | 0.960 | | 2.058 * | | 2.273 | | 2.013 | | 1.733 | | 2.020 † | | 1.784 * |
| 65-69 | 1.309 * | | 1.032 | | 2.690 † | | 3.504 * | | 2.162 | | 1.752 | | 2.250 † | | 1.799 * |
| 70-74 | 1.308 * | | 1.165 | | 3.229 † | | 4.410 † | | 3.572 * | | 2.920 | | 2.962 † | | 2.731 † |
| 75-79 | 1.676 † | | 1.533 * | | 3.736 † | | 5.710 † | | 4.804 † | | 3.926 * | | 3.736 † | | 3.189 † |
| 80-84 | 2.038 † | | 2.016 † | | 4.476 † | | 6.006 † | | 5.160 † | | 3.631 * | | 4.251 † | | 3.381 † |
| 85 and over | 2.745 † | | 3.180 † | | 4.953 † | | 8.823 † | | 8.894 † | | 7.281 * | | 6.476 † | | 6.358 † |
| Female sex (vs male) | 0.887 † | | 0.878 * | | 0.905 | | 0.910 | | 0.789 * | | 1.015 | | 0.782 † | | 0.789 † |
| STEMI as index event (vs NSTEMI) | 0.881 † | | 0.748 † | | 1.118 | | 1.070 | | 0.842 | | 0.807 | | 0.843 † | | 0.810 * |
| PCI or CABG related to index MI | 0.650 † | | 0.617 † | | 0.673 † | | 0.684 † | | 0.502 † | | 0.507 † | | 0.493 † | | 0.506 † |
| Atrial fibrillation | 1.014 | | 1.056 | | 1.408 † | | 1.537 † | | 1.057 | | 1.054 | | 1.143 * | | 1.109 |
| Diabetes mellitus | 1.371 † | | 1.409 † | | 1.257 † | | 1.312 * | | 1.494 † | | 1.535 † | | 1.361 † | | 1.337 † |
| Chronic renal failure | 1.788 † | | 1.811 † | | 1.310 | | 0.991 | | 1.265 | | 1.296 | | 1.762 † | | 1.493 * |
| Dementia/Alzheimer's disease | 0.979 | | 0.832 | | 0.969 | | 0.744 | | 2.029 † | | 2.092 † | | 2.541 † | | 2.972 † |
| Ischemic stroke or TIA | 1.114 * | | 1.228 * | | 4.169 † | | 2.930 † | | 1.601 † | | 1.544 * | | 1.332 † | | 1.227 * |
| Major bleedings | 1.170 * | | 1.264 * | | 1.144 | | 1.206 | | 1.243 | | 1.690 * | | 1.352 † | | 1.517 † |
| Hypertension | 0.910 | | 1.998 * | | 1.319 | | 1.125 | | 0.659 | | 1.355 | | 0.582 † | | 0.630 * |
| Hyperlipidemia | 1.016 | | 0.991 | | 0.974 | | 1.081 | | 0.721 † | | 0.778 | | 0.616 † | | 0.607 † |
| Congestive heart failure | 1.385 † | | 1.474 † | | 1.219 * | | 1.255 * | | 2.000 † | | 1.981 † | | 2.018 † | | 2.119 † |
| Severe liver disease | 1.232 | | 0.607 | | 1.870 | | 1.685 | | 0.721 | | 0.986 | | 1.283 | | 1.229 |
| COPD | 1.156 * | | 1.530 † | | 0.950 | | 1.154 | | 1.842 † | | 1.976 * | | 1.915 † | | 1.908 † |
| Malignancy | 1.160 * | | 1.156 | | 1.272 | | 1.260 | | 0.975 | | 1.165 | | 1.668 † | | 1.683 † |
| SSRI use | 1.008 | | 1.014 | | 1.392 * | | 1.174 | | 0.913 | | 0.913 | | 0.864 | | 0.903 |
| OAP use | 1.084 * | | 1.392 † | | 0.793 * | | 0.980 | | 0.885 | | 1.087 | | 0.675 † | | 0.734 * |
| Abbreviations: CABG, coronary artery bypass grafting; COPD, chronic obstructive pulmonary disease; MI, myocardial infarction; NSTEMI, non-ST-elevation myocardial infarction; OAP, oral antiplatelet; PCI, percutaneous coronary intervention; SSRI, selective serotonin reuptake inhibitor; STEMI, ST-elevation myocardial infarction; TIA, transient ischemic attack | | | | | | | | | | | | | | | |
| * P<0.05 |  |  | |  | |  | |  | |  | |  | |  | |
| † P<0.001 |  |  | |  | |  | |  | |  | |  | |  | |
| The multivariate model simultaneously included all the variables listed in this table. | | | | | | | | | | | | | | | |
